# Molecular Characterization of the *1-Deoxy-D-Xylulose 5-Phosphate Synthase* Gene Family in *Artemisia annua*

**DOI:** 10.3389/fpls.2018.00952

**Published:** 2018-08-02

**Authors:** Fangyuan Zhang, Wanhong Liu, Jing Xia, Junlan Zeng, Lien Xiang, Shunqin Zhu, Qiumin Zheng, He Xie, Chunxian Yang, Min Chen, Zhihua Liao

**Affiliations:** ^1^Key Laboratory of Eco-Environments in Three Gorges Reservoir Region (Ministry of Education), Chongqing Key Laboratory of Plant Ecology and Resources Research in Three Gorges Reservoir Region, SWU-TAAHC Medicinal Plant Joint R&D Centre, School of Life Sciences, Southwest University, Chongqing, China; ^2^School of Chemistry and Chemical Engineering, Chongqing University of Science and Technology, Chongqing, China; ^3^Tobacco Breeding and Biotechnology Research Center, Yunnan Academy of Tobacco Agricultural Sciences, Key Laboratory of Tobacco Biotechnological Breeding, National Tobacco Genetic Engineering Research Center, Kunming, China; ^4^SWU-TAAHC Medicinal Plant Joint R&D Centre, College of Pharmaceutical Sciences, Southwest University, Chongqing, China

**Keywords:** *Artemisia annua*, artemisinin, 1-deoxy-D-xylulose 5-phosphate synthase, gene expression, MEP pathway

## Abstract

*Artemisia annua* produces artemisinin, an effective antimalarial drug. In recent decades, the later steps of artemisinin biosynthesis have been thoroughly investigated; however, little is known about the early steps of artemisinin biosynthesis. Comparative transcriptomics of glandular and filamentous trichomes and ^13^CO_2_ radioisotope study have shown that the 2-C-methyl-D-erythritol-4-phosphate (MEP) pathway, rather than the mevalonate pathway, plays an important role in artemisinin biosynthesis. In this study, we have cloned three *1-deoxy-D-xylulose 5-phosphate synthase* (*DXS*) genes from *A*. *annua* (*AaDXS1*, *AaDXS2*, and *AaDXS3*); the DXS enzyme catalyzes the first and rate-limiting enzyme of the MEP pathway. We analyzed the expression of these three genes in different tissues in response to multiple treatments. Phylogenetic analysis revealed that each of the three *DXS* genes belonged to a distinct clade. Subcellular localization analysis indicated that all three AaDXS proteins are targeted to chloroplasts, which is consistent with the presence of plastid transit peptides in their N-terminal regions. Expression analyses revealed that the expression pattern of *AaDXS2* in specific tissues and in response to different treatments, including methyl jasmonate, light, and low temperature, was similar to that of artemisinin biosynthesis genes. To further investigate the tissue-specific expression pattern of *AaDXS2*, the promoter of *AaDXS2* was cloned upstream of the *β-glucuronidase* gene and was introduced in arabidopsis. Histochemical staining assays demonstrated that *AaDXS2* was mainly expressed in the trichomes of Arabidopsis leaves. Together, these results suggest that *AaDXS2* might be the only member of the DXS family in *A. annua* that is involved in artemisinin biosynthesis.

## Introduction

Terpenoids, also known as isoprenoids, play several important roles in several plant processes. Despite their diverse structures and functions, all terpenoids are derived from the common five-carbon (C_5_) building blocks, isopentenyl diphosphate (IPP) and its isomer dimethylallyl diphosphate (DMAPP). In plants, the C_5_ building blocks are biosynthesized *via* two-independent pathways: cytosolic mevalonate (MVA) pathway that is found in most eukaryotes and 2-C-methyl-D-erythritol-4-phosphate (MEP) pathway that is found in the chloroplasts of photosynthetic eukaryotes and in eubacteria (Querol et al., 2002). Both these pathways are thought to be largely independent. The MVA pathway is primarily responsible for the biosynthesis of sesquiterpenes and triterpenes, whereas the MEP pathway produces precursors for the biosynthesis of major photosynthetic pigments, hormones, and monoterpenes and diterpenes (Dudareva et al., 2005). However, crosstalk between the MVA and MEP pathways occurs during the biosynthesis of some sesquiterpenes, such as artemisinin (Schramek et al., 2009).

The first reaction of the MEP pathway is the condensation of pyruvate with glyceraldehyde-3-phosphate to produce 1-deoxy-D-xylulose 5-phosphate (DXS), which is catalyzed by DXS (Querol et al., 2002). Subsequently, MEP is converted into a 5:1 mixture of IPP and DMAPP via six enzymatic reactions (Rodriguez-Concepcion and Boronat, 2002). Various studies suggest that DXS is a rate-limiting enzyme in the biosynthesis of terpenoids. The expression of *DXS* increases in plant tissues that require high levels of isoprenoids, as exemplified by maize (*Zea mays*; Cordoba et al., 2011), tomato (*Solanum lycopersicum*; Paetzold et al., 2010), glandular trichomes of peppermint (*Mentha piperita*; Lange et al., 1998), and young seedlings of arapdiopsis (*Arabidopsis thaliana*; Estévez et al., 2001). Overexpression or suppression of *DXS* alters the levels of specific isoprenoids in arapdiopsis (Estévez et al., 2001), tomato (Enfissi et al., 2005), and potato (*Solanum tuberosum*; Morris et al., 2006). Thus, DXS is an important target for the manipulation of isoprenoid biosynthesis.

Although most of the enzymes in the MEP pathway are encoded by single copy genes, the DXS enzymes are usually encoded by a small gene family (Rodriguez-Concepcion and Boronat, 2002; Cordoba et al., 2009), which is divided into three distinct phylogenetic clades (Carretero-Paulet et al., 2013). The expression of genes in the different clades varies with development, tissue type, and environmental conditions. The DXS proteins in clade 1, such as DXS1 in arabidopsis (*Cloroplastos alterados 1*, *CLA1*), primarily perform housekeeping functions (Estévez et al., 2000). In contrast, the expression of *DXS* genes in clade 2 is associated with isoprenoid accumulation. For instance, the expression of *MtDXS2* in barrelclover (*Medicago truncatula*) and *ZmDXS2* in maize is correlated with the production of certain apocarotenoids during mycorrhization (Walter et al., 2002; Cordoba et al., 2011). The suppression of these *MtDXS2* results in reduced apocarotenoid accumulation, whereas the accumulation of *MtDXS1* does not affect apocarotenoid production (Walter et al., 2002). Similarly, the expression of *SlDXS2* in tomato exhibits a positive correlation with colonization by mycorrhizal fungi and the accumulation of apocarotenoids (Paetzold et al., 2010). Furthermore, the suppression of *SlDXS2* in tomato leads to a decrease in the accumulation of the monoterpene, β-phellandrene, and an increase in the levels of two sesquiterpenes in leaf trichomes (Paetzold et al., 2010). Likewise, *OsDXS3* in rice (*Oryza sativa*) has been suggested to be involved in defense responses and secondary metabolism (Okada et al., 2007). Together, these findings suggest that the DXS proteins in clade 2 are dedicated to secondary metabolism.

Artemisinin is a sesquiterpene endoperoxide that has been isolated from sweet wormwood (*Artemisia annua*) and extensively used in the treatment of malaria. Artemisinin has received tremendous interest in recent years because of its potential to treat cancer, diabetes, and tuberculosis (Zheng et al., 2017). Remarkable advances have been made in understanding the artemisinin biosynthetic pathway in recent decades. The first committed step in artemisinin biosynthesis is the cyclization of farnesyl diphosphate (FPP) to amorpha-4, 11-diene by amorpha-4, 11-diene synthase (ADS). Through three additional consecutive enzymatic reactions, amorpha-4, 11-diene is converted into dihydroartemisinic acid (DHAA), which is subsequently converted into artemisinin in an enzyme-independent reaction. Additionally, all four artemisinin biosynthesis genes (*ADS*, *CYP71AV1*, *DBR2*, and *ALDH1*) are specifically expressed in glandular secretory trichomes (GSTs), which are 10-cell structures located primarily on the surface of leaves and flower buds in *A. annua* (Chen et al., 2017).

While substantial progress has been made in understanding the later steps of artemisinin biosynthesis, little is known about the MVA and MEP pathways that supply the precursors for artemisinin biosynthesis. It has long been assumed that terpenoid/isoprenoid precursors provided by the MVA pathway are predominantly responsible for artemisinin biosynthesis; however, more recent studies suggest that the MEP pathway also supplies precursors for artemisinin biosynthesis. Either mevinolin (MVA pathway-specific inhibitor) or fosmidomycin (MEP pathway-specific inhibitor) decreases the artemisinin production in treated plants of *A. annua* (Towler and Weathers, 2007). Furthermore, the ^13^CO_2_ study demonstrates that the MEP pathway provides the central isoprenoid unit for the biosynthesis of FPP, which is the substrate for ADS (Schramek et al., 2009). Thus, both pathways are involved in artemisinin biosynthesis; however, genes that encode the enzymes of the MEP and MVA pathways, and the functions of these genes, remain elusive.

Given that the MEP pathway supplies precursors for artemisinin biosynthesis, and the DXS is the rate-limiting enzyme in the MEP pathway, a deeper understanding of the individual *DXS* gene family members will further help in understanding artemisinin biosynthesis and provide new target(s) for manipulating this metabolic pathway. Previously, Graham et al. (2010) have reported two *DXS* genes in *A. annua* with differential expression patterns, based on transcriptome sequencing. In the present work, we performed a detailed analysis of *AaDXS* gene family. Three *AaDXS* genes were cloned, each of which represented a distinct phylogenetic clade. Additionally, comprehensive expression analyses showed that the expression pattern of *AaDXS2* was highly similar to that of artemisinin biosynthesis genes, suggesting that *AaDXS2* is the primary member of the *AaDXS* gene family that is involved in artemisinin biosynthesis.

## Materials and Methods

### Plant Materials and Treatments

Seeds of *A. annua* were collected from the botanical garden of Southwest University, Chongqing, China and stored at 4°C. Seedlings were grown in the greenhouse at 25 ± 1°C under 16 h light/8 h dark photoperiod. For light induction analysis of *DXS* genes, one-month-old seedlings were grown in the dark for 24 h and then shifted to light (Cordoba et al., 2011). Gene expression was examined in the leaves collected at different time points, ranging from 5 min to 12 h; leaves collected at 0 min were used as a control. In order to determine the expression pattern of *DXS* genes during development, the apical bud and the top seven leaves (leaf 1–8, except leaf 3) on the main stem of two-month-old plants were subjected to quantitative real-time polymerase chain reaction (qPCR) analysis (Lu et al., 2013; Czechowski et al., 2016). To analyze the expression of *DXS* genes in response to methyl jasmonate (MeJA), leaves of 2-month-old plants were treated with 300 μM MeJA and harvested at the indicated time points; leaves harvested from plants treated with 0.8% alcohol were used as a control. To study the effect of cold temperature on *DXS* gene expression, *A. annua* seedlings were transferred to the illumination incubator at 4°C (Liu et al., 2017). Subsequently, leaves collected at the indicated time points were subjected to qPCR analysis; leaves harvested at 0 h were used as a control. For analyzing tissue-specific expression profiles of *DXS* genes, five-month-old plants were transferred to 8 h light/16 h dark photoperiod to promote flowering. Flowers, leaves, stems, and roots of *A. annua* plants were collected and used for analyzing tissue expression profiles of *DXS* genes. All treatments performed in this study were replicated three times.

### Cloning of *DXS* Genes and Sequence Analysis

Total RNA was isolated from plant materials using RNAsimple Kit (No. DP419; Tiangen Biotech, Beijing, China) according to the manufacturer’s protocol. Promega M-MLV Kit (Promega, United States) and SMART rapid amplification of cDNA ends (RACE) cDNA Amplification Kit (Clontech, United States) were used for cloning the 3′- and 5′-end of *DXS* cDNAs, respectively. The first-strand 3′- and 5′-RACE-Ready cDNAs were prepared and used as templates for 3′- and 5′-RACE, respectively, according to the manufacturer’s protocol. For sequence analysis, *DXS* amino acid sequences of *A. thaliana* were used as queries to search for *AaDXS* nucleotide sequences in the expressed sequence tag (EST) database of *A. annua* (taxid: 35608) using tBLASTn program. Touchdown PCR was carried out to clone the 3′- and 5′-ends of *AaDXS* genes. Each PCR product was cloned into the pMD-18T vector (Takara, Japan) and sequenced. Subsequently, full-length cDNAs of *AaDXS* genes were amplified by PCR using gene-specific primers (**Supplementary Table S1**). Multiple sequence alignments of *DXS* proteins were performed using Vector-NTI Advance 11.5 software package (Invitrogen, Carlsbad, CA, United States). Phylogenetic tree of *DXS* proteins was constructed with MEGA version 3.0 (Kumar et al., 2004) using the neighbor-joining method with a bootstrap of 1,000 replicates (Saitou and Nei, 1987).

### Subcellular Localization

The putative plastid transit peptides of DXS isoforms were predicted using TargetP (Emanuelsson et al., 2000). Fragments of *AaDXS* genes encoding transit peptides were amplified by PCR using KOD plus (TOYOBO, Japan). Subsequently, all fragments harboring *Sac*I and *Sal*I restriction sites were inserted into the multiple cloning site of pCAMBIA1300-green fluorescent protein (GFP) in-frame with the coding sequence of the GFP gene. Approximately, 20 μg of each plasmid was introduced into mesophyll protoplasts of tobacco (*Nicotiana tabacum*) using polyethylene glycol-mediated transformation (Yoo et al., 2007). GFP fluorescence and chlorophyll autofluorescence were observed using ZEISS LSM700 laser confocal microscope (ZEISS, Germany) at excitation wavelengths of 488 and 555 nm, respectively.

### qPCR Analysis

To investigate the expression of *AaDXS* genes in different tissues as well as under various conditions, qPCR was performed using iQ5 Multicolor Real-Time PCR Detection System (Bio-Rad, United States). The total RNA (2 μg) of *A*. *annua* was used for first-strand cDNA synthesis using GoScript^TM^ Reverse Transcription System (Promega, United States). The qPCR reaction mixtures were prepared with Go*Taq* qPCR Master Mix (Promega, United States), according to the manufacturer’s protocol. PCR amplifications were performed using the following conditions: denaturation at 95°C for 30 s, followed by 40 cycles at denaturation at 95°C for 5 s and annealing and extension at 60°C for 30 s, and a final extension at 72°C for 20 s. Melting curve was used to determine the specificity of amplifications. The *ACTIN* gene was used as reference for normalization of qPCR CT values. Gene-specific primers used for qPCRs were designed using Primer Premier 6 (**Supplementary Table S1**). The 2^-ΔΔCT^ method was used to calculate the relative fold-change in gene expression (Livak and Schmittgen, 2001).

### Promoter Cloning and β-Glucuronidas (GUS) Histochemical Staining

Genomic DNA of *A. annua* was isolated using the CTAB method. A genome walking method, that is, fusion primer and nested integrated PCR (Wang et al., 2011) was carried out to amplify the promoter of *AaDXS2* (*pAaDXS2*). Primers used to amplify *pAaDXS2* are listed in **Supplementary Table S1**. The TSSP software was used to determine the transcription start site of *AaDXS2* (Solovyev and Shahmuradov, 2003). The *cis*-elements in *pAaDXS2* were analyzed using PlantCARE website^1^ and PLACE website^2^.

To investigate the expression pattern of *AaDXS2* in plants, the *pAaDXS2* was cloned into pCAMBIA1391.Z to drive the expression of *GUS* gene. The *pAaDXS2::GUS* construct was introduced into *Agrobacterium tumefaciens* strain GV3101 and transformed into *A. thaliana* by the floral dip method (Zhang et al., 2006). Mature leaves and flowers of 45-day-old arabidopsis seedlings as well as siliques from 2-month-old transgenic *A. thaliana* were used for *GUS* histochemical staining as described previously (Jefferson et al., 1987). *GUS* stained tissues were observed under Olympus SZX16 microscope, and pictures were taken using Olympus DP73 digital camera.

## Results

### The *A. annua* Genome Harbors Three *DXS* Genes

To investigate the *DXS* genes in *A. annua*, tBLASTn program^3^ was used. Amino acid sequences of AtDXS proteins (*At*4G15560, *At*3G21500, and *At*5G11380) were used as queries against the EST database of *A. annua* (taxid: 35608). Three *AaDXS* partial coding sequences (contig16978, contig19217, and contig6280) were obtained. Subsequently, primers for RACE PCRs were designed based on the longest EST sequence. The resulting full-length cDNAs of three *AaDXS* genes were named as *AaDXS1*, *AaDXS2*, and *AaDXS3*.

**FIGURE 1:**
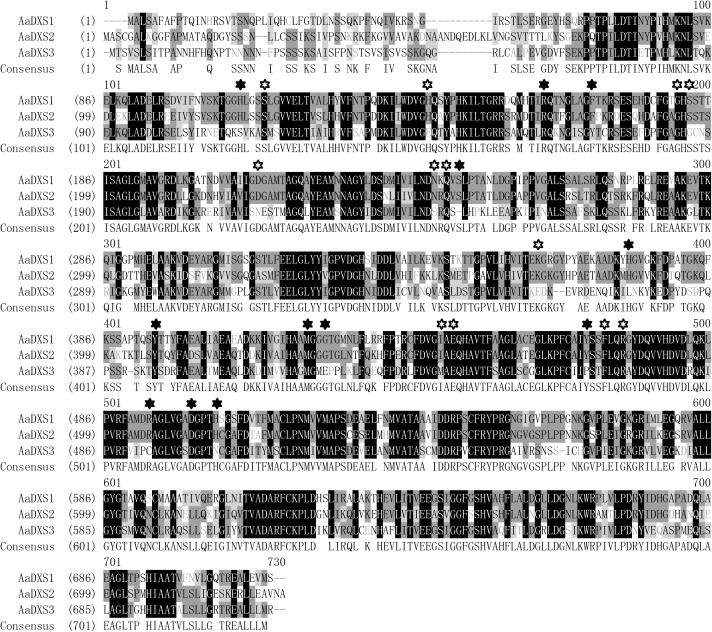
Amino acid sequence alignment of DXS proteins of *Artemisia annua* and *Arabidopsis thaliana*. Solid star indicates the functional residues involved in GAP-binding and hollow star indicates the functional residues involved in TPP-binding.

The full-length *AaDXS1* cDNA was 2,529 bp in length and contained 126 bp 5′ untranslated region (5′UTR), 2,142 bp open reading frame (ORF), and 261 bp 3′UTR (**Supplementary Figure S1**). The *AaDXS2* cDNA consisted of 52-bp 5′UTR, 2,187 bp ORF, and 214 bp 3′UTR (**Supplementary Figure S2**). The *AaDXS3* cDNA was the longest among the three *AaDXS* cDNAs (2,761 bp) and comprised 248 bp 5′UTR and 374 bp 3′UTR (**Supplementary Figure S3**). All three AaDXSs showed features similar to those of known DXS from other plant species, including the presence of an N-terminal targeting sequence, a conserved thiamine diphosphate binding site, and pyridine binding DRAG domain (**Figure 1**).

**FIGURE 2:**
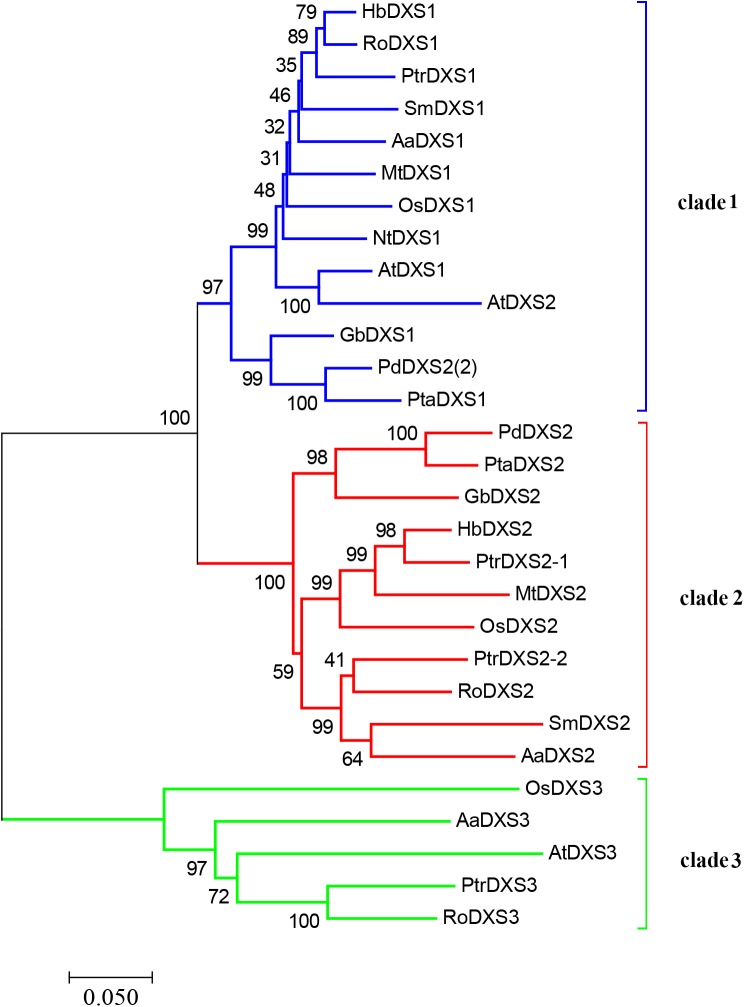
Phylogenetic tree of plant DXS proteins. The tree was constructed with the neighbor-joining method using MEGA program 3.0 with 1,000 bootstrap values. The TAIR or Genebank accession numbers of DXS amino acid sequences used for phylogenetic analysis are as follows: *Arabidopsis thaliana* (DXS1 = *At*4g15560, DXS2 = *At*3g21500, and DXS3 = *At*5G11380); *Ginkgo biloba* (DXS1 = AAS89341.1 and DXS2 = AAR95699.1); *Hevea brasiliensis* (DXS1 = AAS94123.1 and DXS2 = ABF18929.1); *Medicago truncatula* (DXS1 = CAD22530.1 and i = CAN89181.1); *Nicotiana tabacum* (CBA12009.1); *Oryza sativa* (DXS1 = NP_001055524, DXS2 = NP_001059086, and DXS3 = BAA83576); *Pinus densiflora* (DXS1 = ACC54557.1 and DXS2 = ACC54554.1); *Pinus taeda* (DXS1 = ACJ67021.1 and DXS2 = ACJ67020.1); *Populus trichocarpa* (DXS1 = XP_002312717.1, DXS2-1 = XP_002303416.1, DXS2-2 = XP_002331678.1, and DXS3 = XP_002308644.1); *Ricinus communis* (DXS1 = XP_002516843.1, DXS2 = XP_002533688.1, and DXS3 = XP_002514364.1); and *Salvia miltiorrhiza* (DXS1 = ACF21004.1, DXS2 = ACQ66107.1).

A phylogenetic tree was constructed using neighbor-joining method to reveal the evolutionary relationship among the DXS proteins of thirteen plant species, including *A. annua* (**Figure 2**). Phylogenetic analysis showed three clusters of DXS proteins. AaDXS1 grouped in clade 1, which contained well-characterized DXS1 proteins of arabidopsis and *M. truncatula*. AaDXS2 grouped in clade 2 with the well-characterized DXS2 proteins of *M. truncatula* and *Salvia miltiorrhiza*. Partial members within clade 2 play important roles in plant secondary metabolism, especially isoprenoid biosynthesis (Floss et al., 2008; Kai et al., 2012). Compared with clades 1 and 2, clade 3 contained five DXS proteins from *A. annua*, *A. thaliana*, and *O. sativa*. The biological functions of DXS proteins in clade 3 are unclear; however, the DXS proteins in clade 3 are contained at lower expression levels than those in clades 1 and 2 in maize (Cordoba et al., 2011). Studies in arabidopsis and tomato have shown that the DXS proteins in different clades are involved in different biological processes (Paetzold et al., 2010; Carretero-Paulet et al., 2013). Overall, phylogenetic analysis showed that the three *AaDXS* proteins clustered into three distinct clades, suggesting that these three proteins are involved in different biological processes.

### AaDXS Proteins Localize to the Chloroplast

**FIGURE 3:**
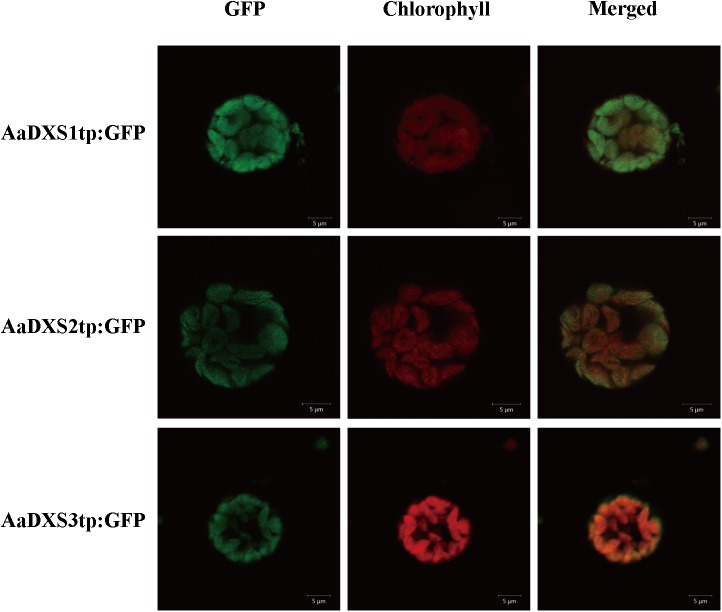
Images of tobacoo mesophyll protoplasts expressing the transit peptides of the *AaDXS* genes fused to the green fluorescent protein (GFP). GFP fluorescence and chlorophyll autofluorescence was detected using confocal microscope.

*In silico* analysis with TargetP 1.1 showed that all three AaDXS proteins carried a plastid transit peptide. To further validate the *in silico* results, nucleotide sequences encoding the putative transit peptides were amplified by PCR and cloned into pCAMBIA1300-GFP vector. These constructs containing the putative transit peptides fused with GFP were then transformed into tobacco mesophyll protoplasts. The GFP signal resulting from the transformation of each vector localized together with the autofluorescence signal of chloroplasts (**Figure 3**). These results demonstrated that all three *Aa*DXS proteins localized to the chloroplast; this is consistent with the plastid localization of the MEP pathway.

### *AaDXS2* and Artemisinin Biosynthesis Genes Show Similar Tissue-Specific Expression

**FIGURE 4:**
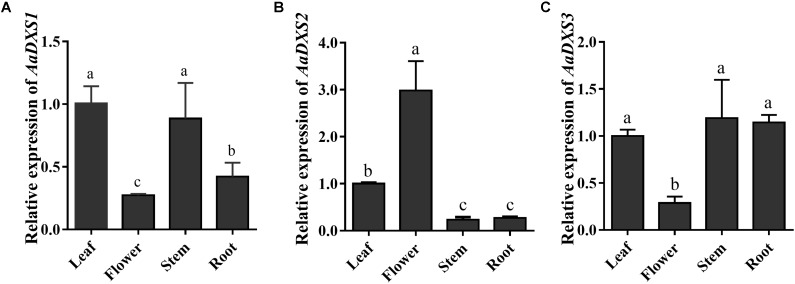
Relative expression levels of *AaDXS1–3* in leaves, flowers, stems, and roots. **(A)** *AaDXS1*; **(B)** *AaDXS2*; and **(C)** *AaDXS3*. Different letters above the bars represent statistically significant differences (*p* < 0.05) detected by Duncan’s multiple test. Data represent mean ± SD of three replicates.

**FIGURE 5:**
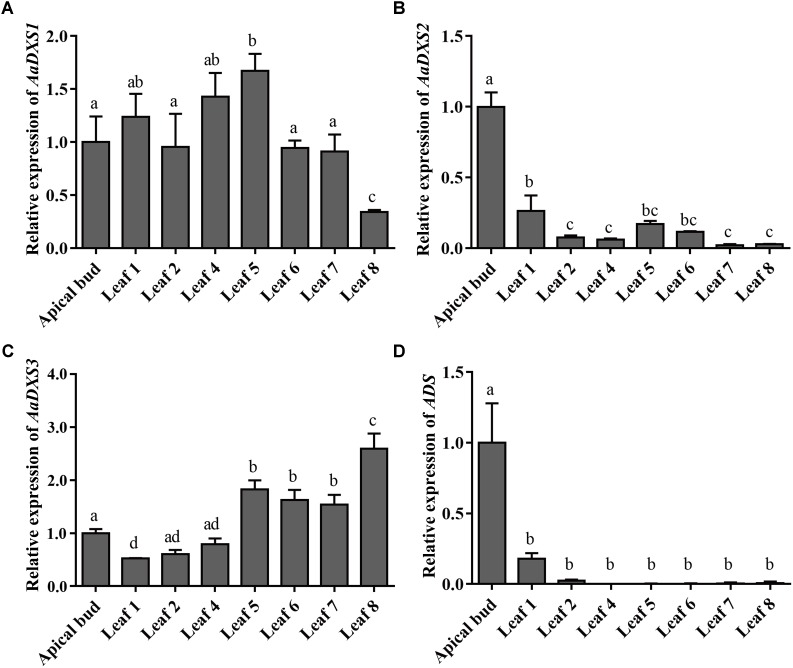
Relative expression levels of *AaDXS1–3* and artemisinin biosynthesis gene, *AaADS*, in the apical bud and in leaves at different positions on the stem. **(A)** *AaDXS1*; **(B)** *AaDXS2*; **(C)** *AaDXS3*; and **(D)** *AaADS*. Different letters above the bars represent statistically significant differences (*p* < 0.05) detected by Duncan’s multiple test. Data represent mean ± SD of three biological replicates.

The expression profiles of *AaDXS* genes were analyzed in different tissues, including flowers, leaves, stems, and roots. Many studies have shown that the artemisinin biosynthesis genes have the highest expression levels in flower buds, followed by leaves, and finally, the roots (Zhang et al., 2015). As shown in **Figure 4**, expression levels of *AaDXS1* and *AaDXS3* were significantly lower in flowers than in other tissues of *A. annua*. However, the expression level of *AaDXS2* in flowers was 3-fold higher than that in leaves and 10-fold higher than that in stems and roots (**Figure 4**). Additionally, the expression of *AaDXSs* was also detected in leaves at different positions on the stem, where the density of glandular trichomes and expression of artemisinin biosynthetic genes show significant difference (Lu et al., 2013). Furthermore, the level of artemisinin precursor, DHAA, progressively declines during leaf maturation (Czechowski et al., 2016). Results of qPCR analysis showed that the expression of *AaDXS2* was high in apical buds, whereas they declined sharply during leaf development (**Figure 5B**). The expression pattern of *AaDXS2* was highly similar to that of the *ADS* gene, which encodes the first enzyme involved in artemisinin biosynthesis (**Figure 5D**). By contrast, the expression of *AaDXS1* was relatively constant in leaves at different positions (**Figure 5A**), and the expression of *AaDXS3* was higher in leaf 8 than in other leaves (**Figure 5C**). Overall, the tissue-specific expression profiles of *AaDXS* genes showed that *AaDXS2* was the only gene whose expression pattern was similar to that of the artemisinin biosynthesis genes, which further indicated that *AaDXS2* was probably highly expressed in glandular trichomes, where artemisinin is biosynthesized.

### *AaDXS2* and Artemisinin Biosynthesis Genes Exhibit Similar Expression Patterns Under Multiple Treatments

**FIGURE 6:**
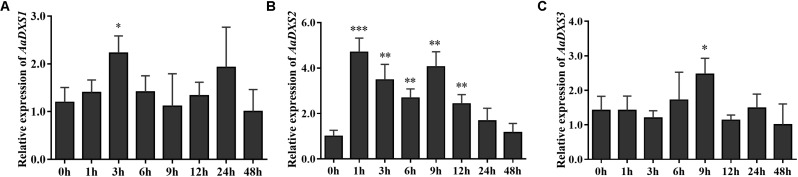
Relative expression levels of *AaDXS1–3* in leaves treated with methyl jasmonate (MeJA). **(A)** *AaDXS1*; **(B)** *AaDXS2*; and **(C)** *AaDXS3*. Statistically significant differences are indicated using asterisks (Student’s *t*-test, ^∗^*p* < 0.1; ^∗∗^*p* < 0.05; and ^∗∗∗^*p* < 0.01). Data represent mean ± SD of three biological replicates.

**FIGURE 7:**
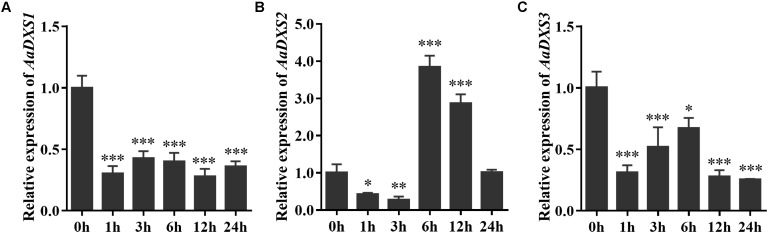
Relative expression levels of *AaDXS1–3* under low temperature (4°C). **(A)** *AaDXS1*; **(B)** *AaDXS2*; and **(C)** *AaDXS3*. Statistically significant differences are indicated using asterisks (Student’s *t*-test, ^∗^*p* < 0.1; ^∗∗^*p* < 0.05; and ^∗∗∗^*p* < 0.01). Data represent mean ± SD of three biological replicates.

Many elicitors and environmental factors, such as MeJA, low temperature, and light, regulate artemisinin biosynthesis (Hao et al., 2017; Liu et al., 2017). The expression of artemisinin biosynthesis genes increases in response to MeJA, cold, and light. To determine which of the three *AaDXS* genes was more important for artemisinin biosynthesis, we analyzed the expression of all three *AaDXS* genes under MeJA, cold, and light treatment. As shown in **Figure 6**, the expression of *AaDXS1* and *AaDXS3* was mildly induced by MeJA treatment at 3 and 9 h (**Figures 6A,C**), whereas that of *AaDXS2* was strongly induced from 1 to 12 h (**Figure 6B**). Under cold treatment, the expression of *AaDXS1* and *AaDXS3* decreased through the time course (**Figures 7A,C**). Although the expression of *AaDXS2* was downregulated at 1 and 3 h under cold stress, its expression was significantly upregulated at 6 and 12 h compared with the control (**Figure 7B**). Additionally, the expression of all three *AaDXS* genes in cold-treated plants was lower than that in the control from 1 to 3 h.

**FIGURE 8:**
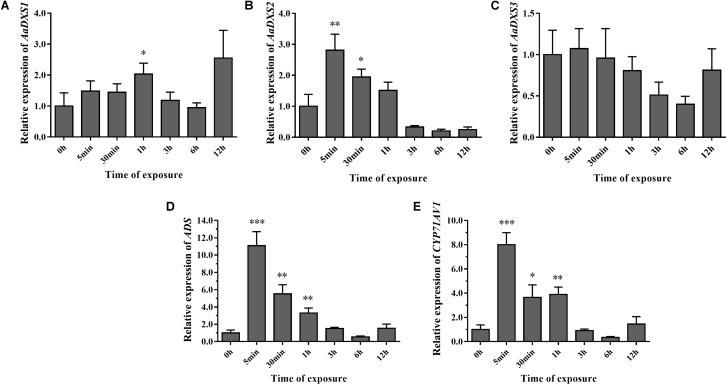
Relative expression levels of *AaDXS1–3* and artemisinin biosynthesis genes (*AaADS*, *AaCYP71AV1*) in leaves exposed to light after 24 h dark treatment. **(A)** *AaDXS1*; **(B)** *AaDXS2*; **(C)** *AaDXS3*; **(D)** *AaADS*; and **(E)** *AaCYP71AV1*. Statistically significant differences are indicated using asterisks (Student’s *t*-test, ^∗^*p* < 0.1; ^∗∗^*p* < 0.05; and ^∗∗∗^*p* < 0.01). Data represent mean ± SD of three biological replicates.

To investigate the effect of light on the expression of *AaDXS* genes, *A. annua* seedlings were placed in a dark room for 24 h and then transferred to the illumination incubator. Leaves were harvested for qPCR analysis at different time points, ranging from 5 min to 12 h. Results of qPCR analysis showed that the expression of *AaDXS1* was slightly induced after 1 h of light exposure (**Figure 8A**); however, no significant differences were detected in the expression of *AaDXS3* throughout the experiment (**Figure 8C**). In contrast, the expression of *AaDXS2* rapidly reached a peak at 5 min and then gradually declined, returning to the control level (**Figure 8B**). Furthermore, expression patterns of *ADS* and *CYP71AV1* under light treatment were similar to that of *AaDXS2* (**Figures 8D,E**).

Overall, the expression analysis of *AaDSX* genes under MeJA, cold, and light treatment demonstrated that among the three *AaDXS* genes, the expression pattern of only *AaDXS2* was similar to that of the artemisinin biosynthesis genes. These data suggest that *AaDXS2* is more important than *AaDXS1* and *AaDXS3* in artemisinin biosynthesis.

### Analysis of AaDXS2 Promoter Activity in Transgenic *A. thaliana*

**FIGURE 9:**
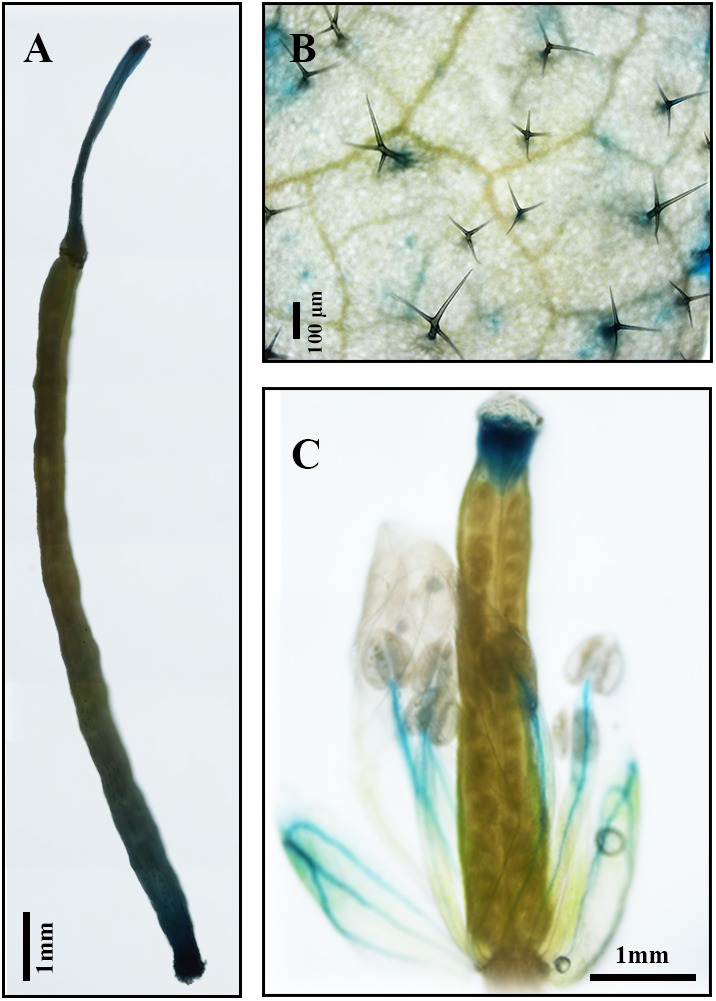
*GUS* histochemical staining of various tissues of *pAaDXS2::GUS* transgenic Arabidopsis plants. The *GUS* reporter gene was driven by the promoter of *AaDXS2* (*pAaDXS2*) and transformed into Arabidopsis. Staining of transgenic **(A)** silique, **(B)** mature leaf, and **(C)** flower is shown.

Results of qPCR analysis showed that the expression of *AaDXS2* in different tissues and under different conditions was similar to that of the artemisinin biosynthesis genes. Subsequently, we cloned the 1,494-bp promoter of *AaDXS2* (*pAaDXS2*; **Supplementary Figure S4**). Sequence analysis of *pAaDXS2* using PLACE and PlantCARE revealed the presence of several light-responsive elements, such as Box I and GATA-motifs; MeJA-responsive elements, such as CGTCA- and TGACG-motifs; and stress-responsive elements, such as Box-W1 that responds to fungal elicitors, HSE involved in high temperature stress, and TC-rich repeats associated with plant defense and stress. To investigate the cellular compartmentalization of *AaDXS2*, we cloned *pAaDXS2* upstream of the *GUS* reporter gene and transformed the *pAaDXS2::GUS* construct in *A. thaliana*. Different organs of 45-day-old transgenic arabidopsis plants, including mature leaves, flowers, and siliques, were used for GUS histochemical staining. GUS staining was observed in the stigma, stamen, and pedicel but not in petals and young carpel (**Figures 9A–C**). More importantly, strong GUS staining was observed in the trichomes of mature leaves (**Figure 9B**).

## Discussion

The MEP pathway provides structural molecules for the synthesis of numerous key metabolites, such as terpenoids, phytohormones, chlorophyll, and carotene. Understanding this biosynthetic route is important for modulating the production of key isoprenoids. Although most of the enzymes in the MEP pathway are encoded by single-copy genes (Rodriguez-Concepcion and Boronat, 2002; Cordoba et al., 2009), the first key enzyme, DXS, is encoded by a small gene family composed of 2–4 genes in plants, such as arabidopsis (Carretero-Paulet et al., 2013), alfalfa (*Medicago sativa*; Walter et al., 2002), and maize (Cordoba et al., 2011). Souret et al. (2002) reported a *DXS* gene, named *DXSPS*, in *A. annua*; the nucleotide sequence of *AaDXSPS* was 98.7% similar to that of *AaDXS1* cloned in this study (**Supplementary Figure S5**). Thus, we propose that *DXPS* and *AaDXS1* represent the same gene. However, transcript levels of *AaDXS1* only showed a slight increase under light treatment, whereas those of *DXPS* significantly increased in root cultures grown under continuous light compared with those grown in the dark (Souret et al., 2002). This discrepancy might be due to the different plant material used in these experiments; Souret et al. (2002) performed expression analysis in *A. annua* root cultures, whereas we used leaves in this study. Previously, 454 sequencing of EST libraries in *A. annua* have revealed two DXS contigs (EZ216572 and EZ167196; Graham et al., 2010). Aligning these two contigs with the *AaDXS* genes cloned in this study showed that EZ216572 and EZ167196 represent partial coding sequences of *AaDXS1* and *AaDXS2*, respectively. Moreover, transcriptome data showed that the expression of EZ167196 was significantly higher in the GSTs of flower buds and young leaves than in those of mature leaves and young leaf meristem. In contrast, EZ216572 showed higher expression levels in the GSTs of young leaves and in cotyledons. These data are consistent with the expression analysis results of this study, showing that *AaDXS2* was mainly expressed in young leaves and flower buds.

Several studies suggest that different genes in the *DXS* family are responsible for the biosynthesis of different isoprenoids (Enfissi et al., 2005; Paetzold et al., 2010; Cordoba et al., 2011). Henceforth, it is important to investigate the roles of different DXS isoforms in the biosynthesis of specific metabolites. Although enzymes involved in artemisinin biosynthesis have been determined, little is known about the enzymes involved in the MEP pathway. In the present study, we cloned three *AaDXS* genes, each of which encoded plastid-localized proteins. Expression analysis of *AaDXS* genes indicated that the expression pattern of *AaDXS2* was similar to that of the artemisinin biosynthesis genes. The analysis of *AaDXS2* promoter in transgenic arabidopsis demonstrated that *AaDXS2* was mainly expressed in trichomes.

Amino acid sequence alignment revealed that the three *AaDXS* proteins shared 42.3% identity. Moreover, low conservation in the TPP and GAP binding sites of *AaDXS3* suggested that *AaDXS3* diverged from *AaDXS1* and *AaDXS2*. The relatively low similarity in DXS protein sequences implies the functional divergence of DXS family members that have been shown previously (Cordoba et al., 2011). Phylogenetic analysis showed that *AaDXS1* clustered with *AtDXS1* and soybean (*Glycine max*) DXS1 in clade 1; it has been shown that the function of *AtDXS1* and *GmDXS1* is related to chloroplast development and chlorophyll synthesis (Mandel et al., 1996; Zhang et al., 2009). Most of the DXS enzymes involved in the biosynthesis of secondary metabolites in plants belonged to clade 2. For example, *MtDXS2*, which plays an important role in apocarotenoid biosynthesis, groups in clade 2 (Floss et al., 2008). The expression of *DXS2* in hairy roots of red sage (*Salvia miltiorrhiza*) is positively correlated with the accumulation of tanshinones (Kai et al., 2012). Although the biological functions of DXS proteins in clades 1 and 2 in plants are relatively clear, those of DXS proteins in clade 3 are still unclear. The expression of *ZmDXS3* is lower than that of the other DXS isogenes in maize, by which we predicted that *ZmDXS3* might be involved in the biosynthesis of derivatives from the MEP pathway at a lower level, such as plant hormones (Cordoba et al., 2009).

Because artemisinin biosynthesis genes are specifically expressed in glandular trichomes, transcripts of artemisinin biosynthesis genes are accumulated to the highest levels in flowers followed by young leaves, as these tissues have more glandular trichomes than the other plant tissues (Lu et al., 2013). In this study, we showed that the expression of both *AaDXS1* and *AaDXS3* was lower in flowers than in other tissues, whereas that of *AaDXS2* was the highest in flowers. Additionally, the expression level of *AaDXS2* in leaves at different positions on the stem was similar to that of the artemisinin biosynthesis genes. This differential expression of *AaDXS2* is consistent with the density of glandular trichomes, which is the highest in the apical bud and decreases in leaves with an increasing developmental age (Lu et al., 2013). Results showed that *AaDXS2* was mainly expressed in the glandular trichomes of leaves, which was consistent with the *GUS* staining patterns observed in transgenic *A. thaliana* (**Figure 9B**). In addition to trichomes of mature leaves, *GUS* staining of transgenic arabidopsis showed that *AaDXS2* was also expressed in the stigma, stamen, and silique. This result was similar to the expression analysis of *AtDXS2* in arabidopsis in which transcripts were detected at highest levels in siliques and inflorescences (Carretero-Paulet et al., 2013). We hypothesized that this might be due to DXS, which is a rate-limiting enzyme in MEP pathway, and which is responsible for biosynthesis of many isoprenoids. *SlDXS2* plays an important role in isoprenoid biosynthesis and trichome development in tomato. The suppression of *SlDXS2 via* RNAi in tomato results in a decrease in the level of the monoterpene, β-phellandrene, and an increase in trichome density (Paetzold et al., 2010). *AaDXS2* might be responsible for the biosynthesis of other isoprenoids excepted to artemisinin. Notably, among the three *DXS* genes of *A. annua*, only *AaDXS2* showed tissue-specific expression patterns that were similar to those of artemisinin biosynthesis genes, suggesting that *AaDXS2* might play a more important role in biosynthesis of artemisinin.

Generally, the *DXS* genes belonging to clade 1 exhibit constitutive expression in plants. For example, *PaDXS1* is ubiquitously expressed in various tissues and is not induced by elicitors (Phillips et al., 2007). Nevertheless, many *DXS* genes in clade 2 exhibit higher expression in response to various elicitors, such as MeJA and light (Cordoba et al., 2011; Kai et al., 2012). Previous studies have shown that MeJA is a powerful elicitor of artemisinin biosynthesis genes; its application induces the expression of artemisinin biosynthesis genes, resulting in a greater accumulation of artemisinin in MeJA-treated plants (Wang et al., 2010; Caretto et al., 2011; Xiang et al., 2015). The strong induction of *AaDXS2* expression following MeJA treatment observed in this study is consistent with the expression of artemisinin biosynthesis genes under MeJA treatment shown previously (Gong et al., 2006; Phillips et al., 2007). Cold stress is effective in enhancing artemisinin biosynthesis via two-independent pathways: converting DHAA into artemisinin (Wallaart et al., 2000) and promoting endogenous MeJA biosyntheis (Liu et al., 2017). Among the three *AaDXS* genes, the expression of *AaDXS1* and *AaDXS3* was inhibited under cold stress, whereas that of *AaDXS2* was significantly increased.

In *A. thaliana*, the MEP pathway genes, except HDR, are upregulated by light, suggesting that light is an important regulator of the MEP pathway in plants (Hsieh and Goodman, 2005). Overexpression of *At*CRY1, a blue light receptor, in *A. annua* promotes artemisinin accumulation, leading to the conclusion that light is a vital factor in regulating artemisinin biosynthesis (Hong et al., 2009). This conclusion is corroborated by a recent study (Hao et al., 2017). The expression of artemisinin biosynthesis genes (*ADS*, *CYP71AV1*, *DBR2*, and *ALDH1*) is upregulated under light. Furthermore, JA-induced artemisinin biosynthesis is dependent on light. Consistent with the study of Hao et al. (2017), we showed that the expression of *ADS* and *CYP71AV1* increased under light. More importantly, only *AaDXS2* was induced rapidly and dramatically after light exposure; this expression pattern of *AaDXS2* was similar to that of the artemisinin biosynthesis genes. Similarly in maize, the expression pattern of *ZmDXS1* and *ZmDXS2* were increased in plants exposed to light treatment (Cordoba et al., 2011). Additionally, the expression pattern of *AaDXS2* was similar with *ADS* and *CYP71AV1* in *A. annua*, indicating that *Aa*DXS2 is involved in the light-mediated regulation of artemisinin biosynthesis.

## Conclusion

In conclusion, this study reports a detailed analysis of the expression profiles of three *DXS* genes in different tissues of *A. annua*. Additionally, we have shown that elicitors, such as MeJA, and various environmental factors modulate the expression of *AaDXS* genes. The expression of *AaDXS2* was very similar to that of the artemisinin biosynthesis genes, suggesting that *Aa*DXS2 might be the only DXS isoform involved in the biosynthesis of artemisinin. This work extends the analyses of *DXS* genes in other plants and provides a new potential target for the modulation of artemisinin production.

## Author Contributions

FZ and ZL conceived and designed the study. FZ, WL, and JX performed the RNA isolation, qRT-PCR analysis, and plant transformation. JZ, HX, and LX performed the subcellular localization and GUS staining. SZ, QZ, and CY managed *Artemisia annua* and Arabidopsis. MC analyzed the data. ZL prepared the manuscript. All authors have read and approved the manuscript.

## Conflict of Interest Statement

The authors declare that the research was conducted in the absence of any commercial or financial relationships that could be construed as a potential conflict of interest.

## Footnotes

1 http://bioinformatics.psb.ugent.be/webtools/plantcare/html/

2 http://www.dna.affrc.go.jp/PLACE/

3 https://blast.ncbi.nlm.nih.gov/Blast.cgi

## Supplementary Material

FIGURE S1 The cDNA sequence of *AaDXS1*.

FIGURE S2 The cDNA sequence of *AaDXS2*.

FIGURE S3 The cDNA sequence of *AaDXS3*.

FIGURE S4 The putative *cis*-elements on the promoter of *AaDXS2*.

FIGURE S5 Amino acidsequence alignment of *AaDXS1* and *DXPS* (AF182286.2).

TABLE S1 The primers used in this study.
