# Supplementary figures and images for: Molecular Characterization of the 1-Deoxy-D-Xylulose 5-Phosphate Synthase Gene Family in Artemisia annua
Source: Front Plant Sci. 2018 Aug 2;9:952. doi: 10.3389/fpls.2018.00952 (PMC6084332; doi:10.3389/fpls.2018.00952)

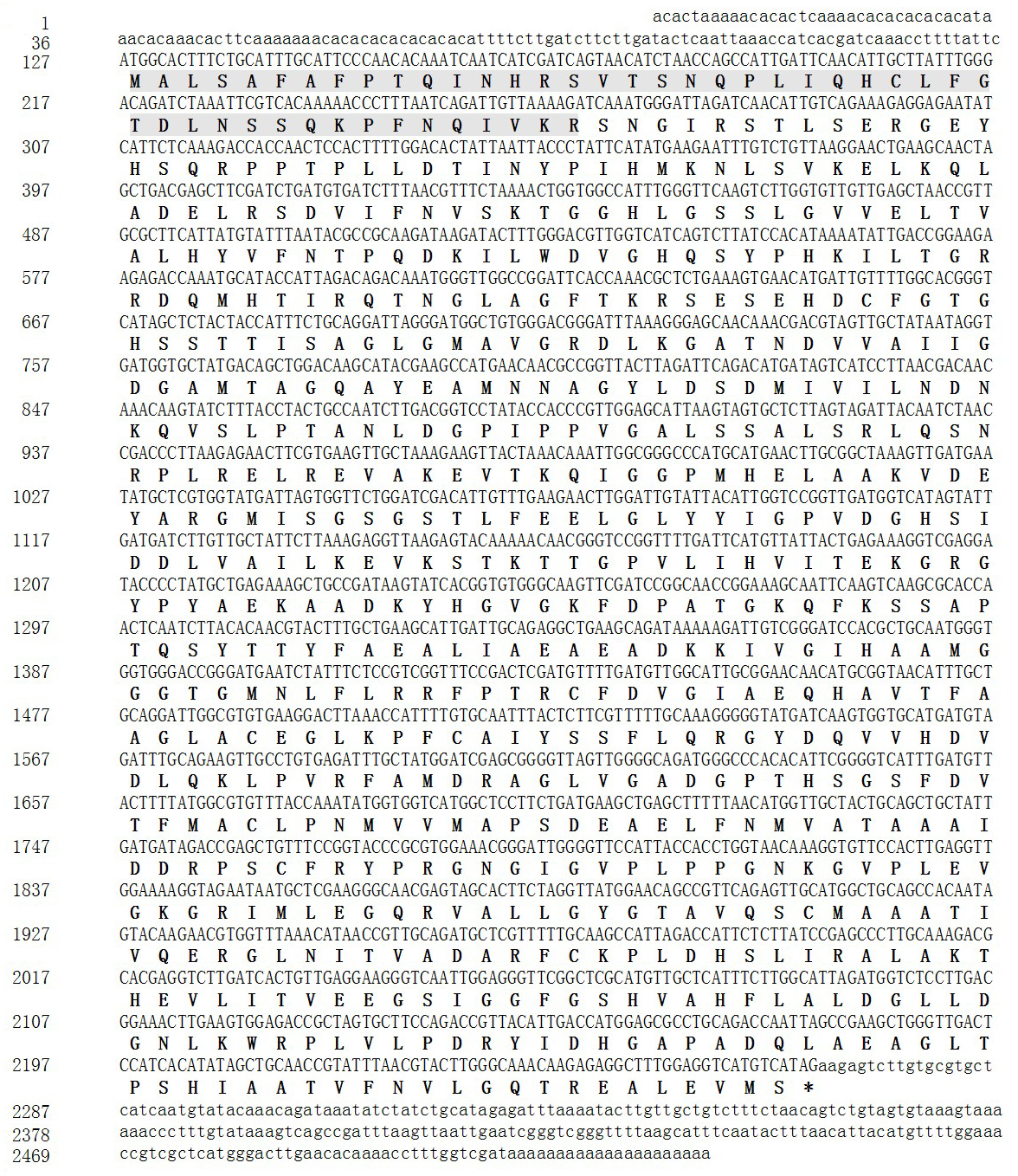

Supplement: FIGURE S1 — The cDNA sequence of AaDXS1. [file Image_1.JPEG]

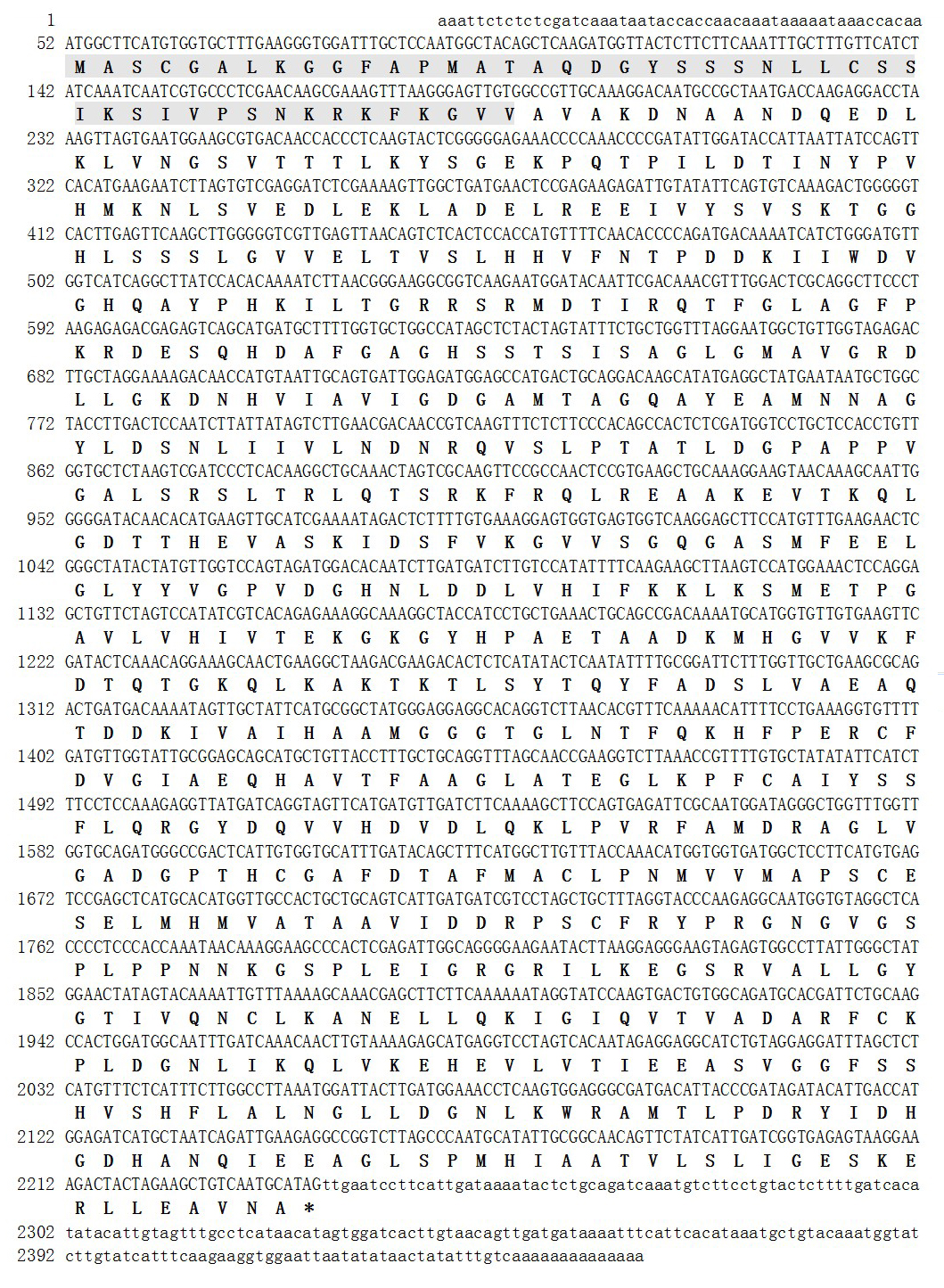

Supplement: FIGURE S2 — The cDNA sequence of AaDXS2. [file Image_2.JPEG]

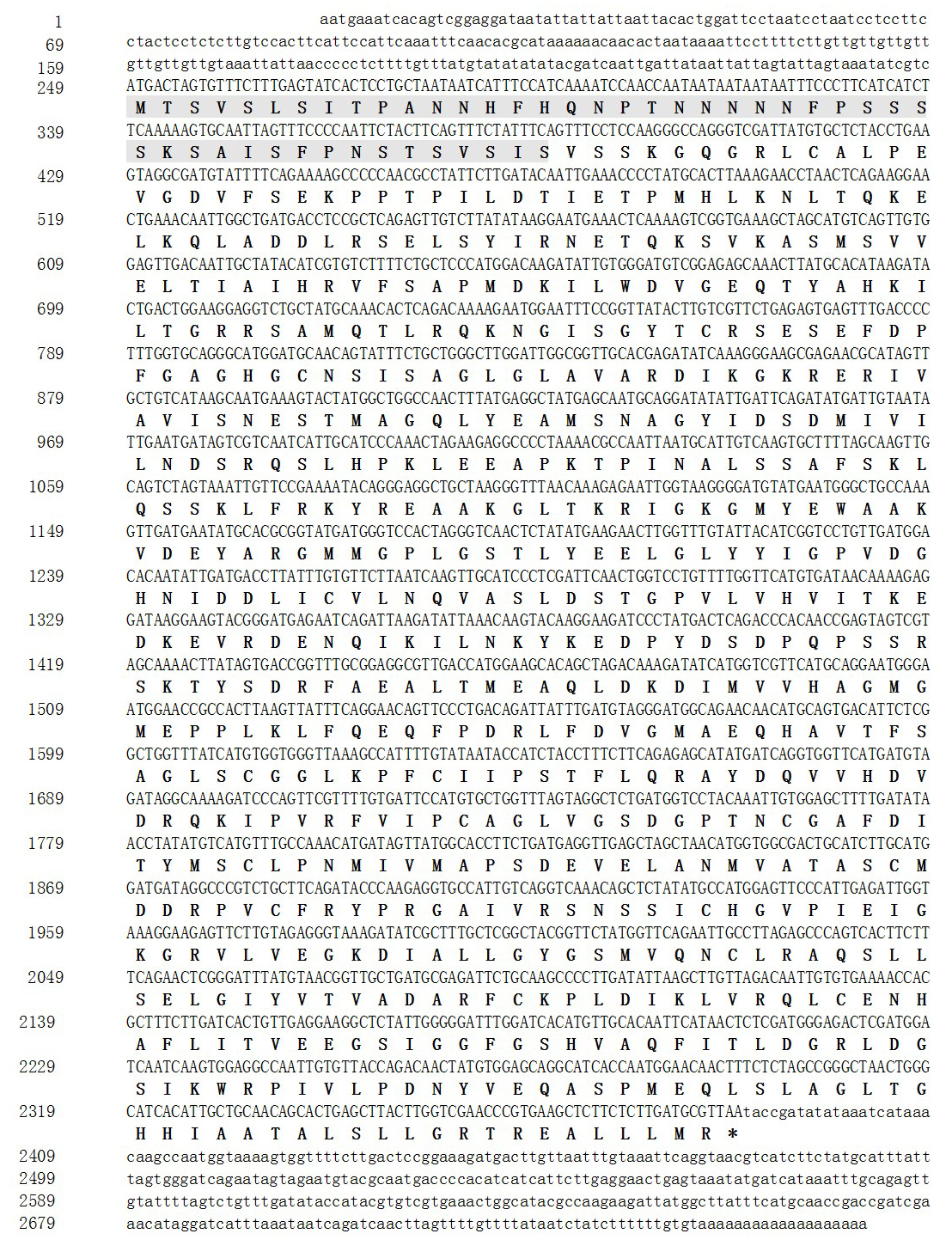

Supplement: FIGURE S3 — The cDNA sequence of AaDXS3. [file Image_3.JPEG]

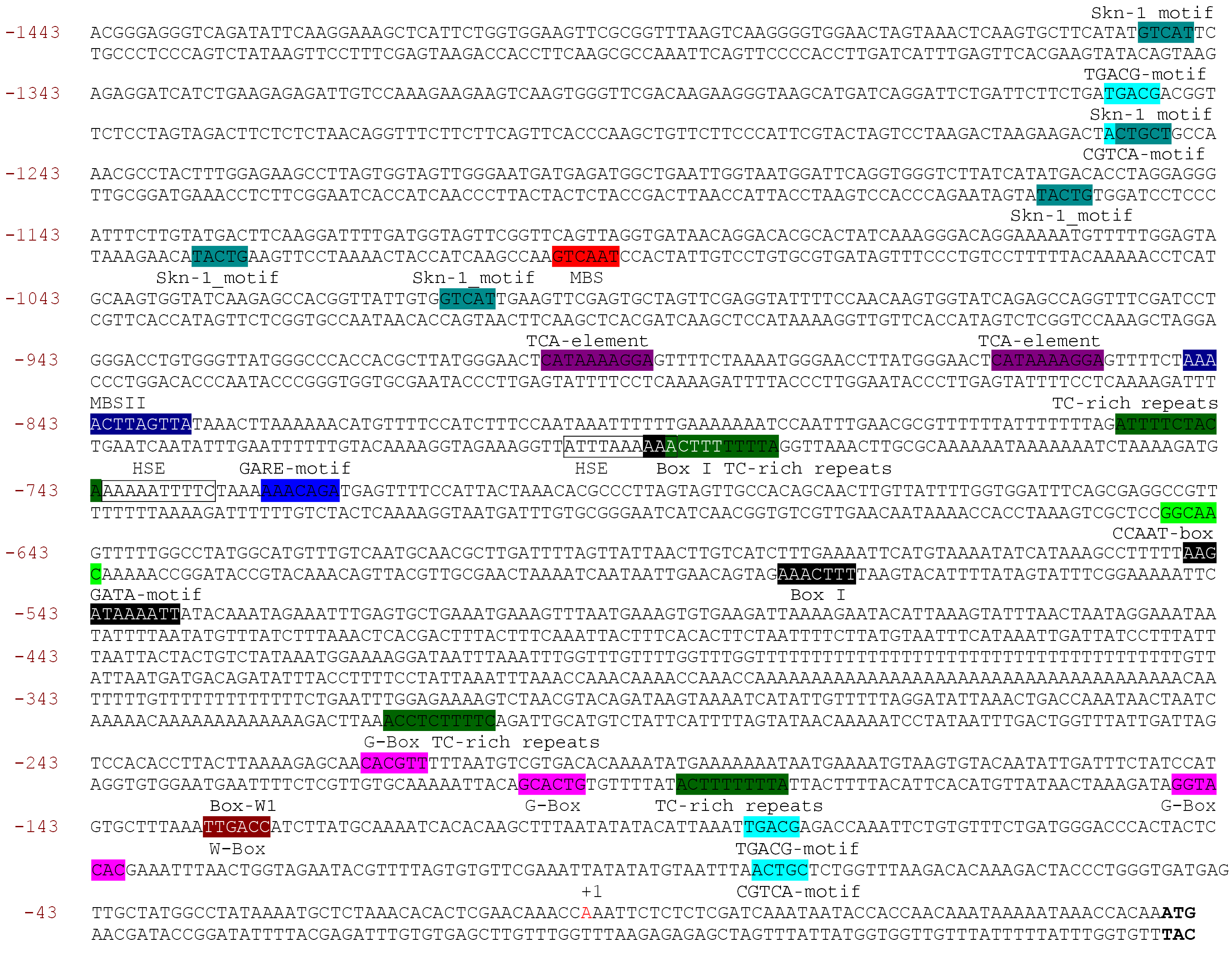

Supplement: FIGURE S4 — The putative cis-elements on the promoter of AaDXS2. [file Image_4.JPEG]

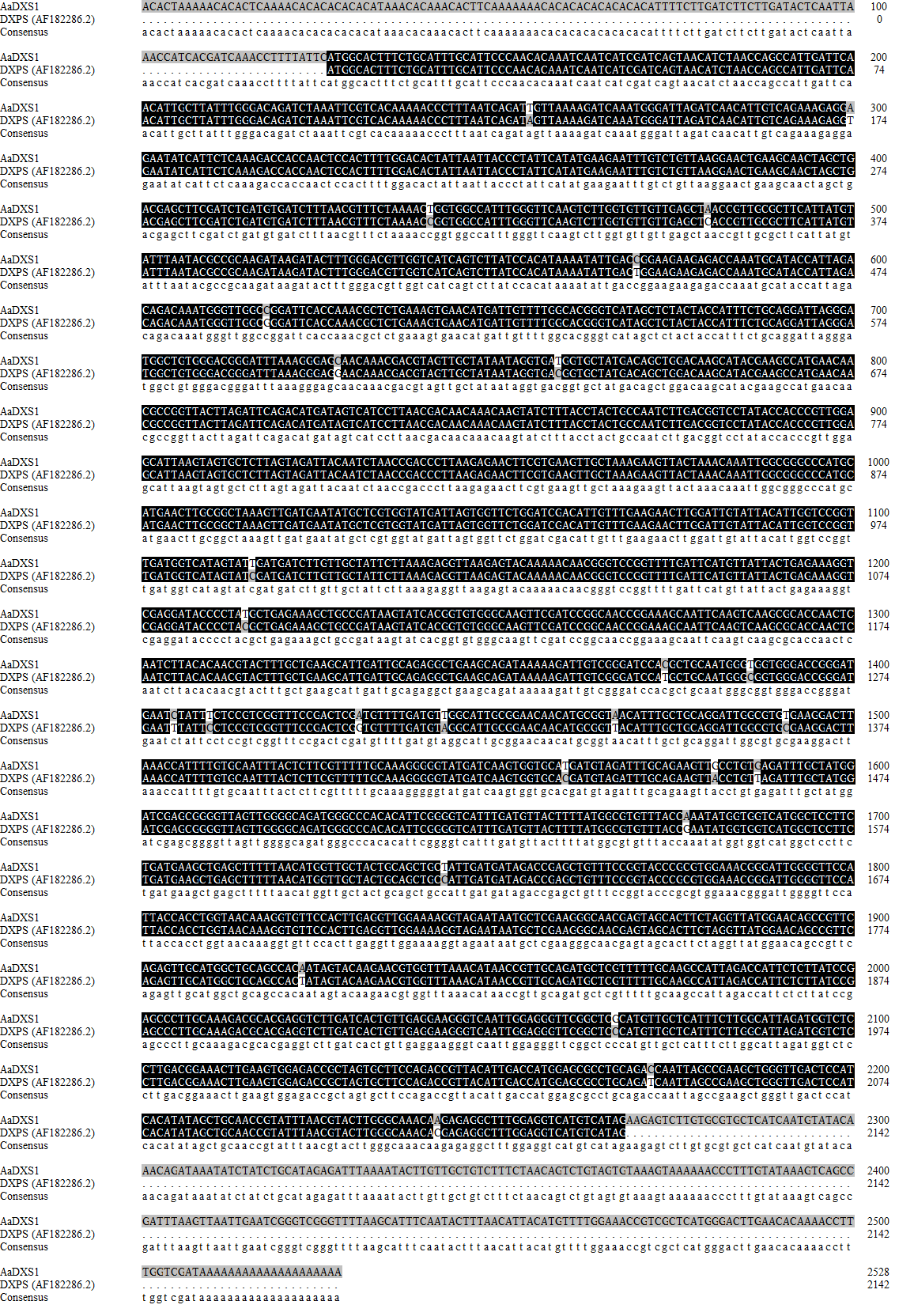

Supplement: FIGURE S5 — Amino acidsequence alignment of AaDXS1 and DXPS (AF182286.2). [file Image_5.TIF]
