# Supplementary material for: Molecular Characterization of the 1-Deoxy-D-Xylulose 5-Phosphate Synthase Gene Family in Artemisia annua
Source: Front Plant Sci. 2018 Aug 2;9:952. doi: 10.3389/fpls.2018.00952 (PMC6084332; doi:10.3389/fpls.2018.00952)
Supplement: TABLE S1 — The primers used in this study. [file Table_1.DOCX]

| **Primers for DXSs 3’ RACE** | |
| --- | --- |
| Primer name | Sequence 5’-3’ |
| **DXS1-3-1** | 5’- ATTGGCGTGCGAAGGACTTAAACC-3’ |
| **DXS1-3-2** | 5’- AGCAGGGTTAGTTGGGGCAGATG-3’ |
| **DXS2-3-1** | 5’- GAGGCATCTGTAGGAGGATTTAG -3’ |
| **DXS2-3-2** | 5’- GCGATGACATTACCCGATAGA -3’ |
| **DXS3-3-1** | 5’-GAAGGCTCTATTGGGGGATTTGG-3’ |
| **DXS3-3-2** | 5’-GCTAACTGGACATCACATTGCCGC-3’ |
| **Cloned AMV RT Module** | 5’-GCTGTCAACGATACGCTACGTAACGGCATGACAGTGTTTTTTTTTTTT  TTTTTT -3’ |
| **GeneRacer 3'Primer** | 5’- GCTGTCAACGATACGCTACGTAACG -3’ |
| **GeneRacer 3'Nested Primer** | 5’- CGCTACGTAACGGCATGACAGTG -3’ |

| **Primers for DXSs 5’ RACE** | |
| --- | --- |
| Primer name | Sequence 5’-3’ |
| **DXS1-5’-1** | 5’- TAAATCCCGTCCCACAGCCATCCC-3’ |
| **DXS1-5’-2** | 5’- GGAGTTGGTGGTCTTTGAGAATGA -3’ |
| **DXS2-5’-1** | 5’- GCTGTGGGAAGAGAAACTTGACGGT -3’ |
| **DXS2-5’-2** | 5’- CGTCTCTCTTAGGGAAGCCTGCGA -3’ |
| **DXS3-5’-1** | 5’- AGCAACCGTTACATAAATCCCGAGT-3’ |
| **DXS3-5’-2** | 5’- CCATAGAACCGTAGCCGAGCAAAGC-3’ |
| **UPM-long** | 5’-CTAATACGACTCACTATAGGGCAAGCAGGGGT  ATCAACGCAGAGT-3’（Long） |
| **UPM-Short** | 5’-CTAATACGACTCACTATAGGGC-3’（Short） |
| **NUP** | 5’-AAGCAGGGGTATCAACGCAGAGT-3’ |

| **Primers for DXSs transpeptid cloing** |
| --- |

| Primer name | Sequence 5’-3’ |
| --- | --- |
| **DXS1-Ftp1** | 5-C*GAGCTC*ATGGCACTTTCTGCATTTGC-3 |
| **DXS1-Ftp1** | 5-GC*GTCGAC*TCTTTTAACAATCTGATTAAAG-3 |
| **DXS2-Ftp2** | 5-C*GAGCTC*ATGGCTTCATGTGGTGCTTTG-3 |
| **DXS2-Rtp2** | 5-GC*GTCGAC*CACAACTCCCTTAAACTTTC-3 |
| **DXS3-Ftp3** | 5-C*GAGCTC*ATGACTAGTGTTTCTTTGAG-3 |
| **DXS3-Rtp3** | 5-GC*GTCGAC*TGAAATAGAAACTGAAGTAG-3 |

| **Primers for *AaDXS2* promoter cloning** |
| --- |

| Primer name | Sequence 5’-3’ | Usage |
| --- | --- | --- |
| **FP1** | GTAATACGACTCACTATAGGGCACGCGTGGTNTCGASTWTSGWGTT | 1st PCR primer |
| **FP2** | GTAATACGACTCACTATAGGGCACGCGTGGTNGTCGASWGANAWGAA | 1st PCR primer |
| **FP3** | GTAATACGACTCACTATAGGGCACGCGTGGTWGTGNAGWANCANAGA | 1st PCR primer |
| **FP4** | GTAATACGACTCACTATAGGGCACGCGTGGTAGWGNAGWANCAWAGG | 1st PCR primer |
| **FP5** | GTAATACGACTCACTATAGGGCACGCGTGGTNGTAWAASGTNTSCAA | 1st PCR primer |
| **FP6** | GTAATACGACTCACTATAGGGCACGCGTGGTNGACGASWGANAWGAC | 1st PCR primer |
| **FP7** | GTAATACGACTCACTATAGGGCACGCGTGGTNGACGASWGANAWGAA | 1st PCR primer |
| **FP8** | GTAATACGACTCACTATAGGGCACGCGTGGTGTNCGASWCANAWGTT | 1st PCR primer |
| **FP9** | GTAATACGACTCACTATAGGGCACGCGTGGTNCAGCTWSCTNTSCTT | 1st PCR primer |
| **FSP1** | GTAATACGACTCACTATAGGGC | 2nd PCR primer |
| **FSP2** | ACTATAGGGCACGCGTGGT | 3rd PCR primer |
| **W1DXS2sp1** | TGCTGCTCCGCAATACCAACAT | 1st PCR primer |
| **W1DXS2sp2** | GTGTTAAGACCTGTGCCTCCTCC | 2nd PCR primer |
| **W1DXS2sp3** | GTCTTCGTCTTAGCCTTCAGTTGC | 3rd PCR primer |
| **W2DXS2sp1** | ATAGCCCGAGTTCTTCAAACATGGA | 1st PCR primer |
| **W2DXS2sp2** | GCTCCTTGACCACTCACCACTCC | 2nd PCR primer |
| **W2DXS2sp3** | CTTAGCCATGATTGTCTTGTAACC | 3rd PCR primer |

| **Primers for qRT-pCR** | |
| --- | --- |
| ***DXS1-qF*** | AGTGCTTCCAGACCGTTACATTG |
| ***DXS1-qR*** | GCCTCTCTTGTTTGCCCAAG |
| ***DXS2-qF*** | GCAACTGAAGGCTAAGACGAAGACA |
| ***DXS2-qR*** | TGCTGCTCCGCAATACCAACATC |
| ***DXS3-qF*** | CCAAGAGGTGCCATTGTCAGGTC |
| ***DXS3-qR*** | ACCATAGAACCGTAGCCGAGCAA |
| ***ADS-qF*** | AATGGGCAAATGAGGGACAC |
| ***ADS-qR*** | TTTCAAGGCTCGATGAACTATG |
| ***CYP71AV1-qF*** | CACCCTCCACTACCCTTG |
| ***CYP71AV1-qR*** | GACACATCCTTCTCCCAGC |
| ***ACTIN-qF*** | CCATTGAACACGGTATTG |
| ***ACTIN-qR*** | AGGAACATTGAAGGTCTC |
